# Determinants of neonatal mortality among newborns admitted in neonatal intensive care unit at Dilla University Referral Hospital in Gedeo Zone, Southern, Ethiopia: unmatched case control study

**DOI:** 10.1186/s12887-021-02780-3

**Published:** 2021-07-08

**Authors:** Atnafu Adem, Azmach Dache, Aregahegn Dona

**Affiliations:** 1 Loka Abaya District Health, Sidama Regional State Hantate, Ethiopia; 2 Social and Population Health Department, Yirgalem Hospital Medical College, Sidama Yirgalem, Ethiopia

**Keywords:** Neonatal mortality, Determinants, Southern Ethiopia

## Abstract

**Background:**

Around two and half million neonatal mortality occurred in 2017, especially in developing countries. This study was conducted to determine the determinants of neonatal mortality among newborns admitted in the neonatal intensive care unit at Dilla University Referral Hospital in Gedeo Zone, Southern Ethiopia.

**Methods:**

An unmatched case-control study was conducted from February, 24 to March 6, 2020 at Dilla University Referral Hospital in Gedeo Zone Southern Ethiopia. A total of 304 neonates (76 cases and 228 controls) were involved. Neonates registered as died were considered as cases and neonates registered as improved were considered as controls. Data were extracted by pretested checklists from medical records of neonates admitted during the last one year period. Data was entered into EpiData3.1, and analyzed by statistical package for social science software Version 22.Bivariate and multivariate logistic regressions were used to identify determinants associated with neonatal mortality. Finally, AORs at 95 % CI and P-values < 0.05 were used to declare statistical significance.

**Results:**

In this study, a total of 304 cases were assessed with 100 % reviewed rate. It was found that referrals from other health facilities, [AORs = 2.43, 95 % CI (1.14, 5.22)], gestational age < 37 weeks,[AORs = 2.50, 95 % CI (1.12, 5.58)], the weight of newborn < 2500 g, [AORs = 2.44, 95 % CI (1.13, 5.28)], neonates positive for sepsis, [AORs = 2.45, 95 % CI (1.11, 5.41)]and neonates who not breastfed within first hour after delivery,[AORs = 5.24, 95 % CI (2.42, 11.37)] were statistically significant determinants to neonatal mortality.

**Conclusions:**

This study suggests that referral, gestational age, weight of newborn, sepsis and breastfeeding were significant determinants to neonatal mortality. This study shows that neonatal intensive care unit service should be strengthened in Dilla University Referral Hospital; targeting neonate aged below 28 days. Most of these determinants may be prevented and minimized by strengthening referral linkage, improving intrapartum and postpartum care.

## Introduction

Neonatal mortality is defined as the death of live-born within the first 28 days of life. Neonatal mortality can be subdivided into very early, early and late neonatal mortality. The very early and early neonatal mortalities are the deaths occurring on the first day and within the first seven days of live-born. Whereas the deaths happening after the seventh days but before the 28 day of life considered as late neonatal mortality [1, 2]. Neonatal mortality rate (NMR) is the number of neonatal deaths per 1000 live births in a given year and is a key indicator of the health of population [1, 3, 4]. The main causes for neonatal mortality are related to congenital anomalies, preterm, small for gestational age, intrapartum injuries, infections, low birth weight, and challenges of adapting extra uterine environment [5].

At the global level, around two and half million neonatal mortality happened within four weeks of life in 2017. About 36 % of them died within the first day they were born, and 75 % of all neonatal mortality occurred in the first week of life. Almost all mortalities of neonates were registered in growing countries and it becomes the riskiest time for an under-five survival [6]. Two-thirds of all live-born death is contributed from 12 countries, six of them are in Sub-Saharan Africa countries and they accounted for about 60 % of all neonatal mortalities [6, 7].In Ethiopia, about 87,000 neonates die each year within 28 days of life after birth and therate of mortality is among the highest in the world [8]. The trend of mortalities also increased from 29 in Ethiopian Demographic Health Survey (EDHS2016) to 30 in Ethiopian Min Demographic Health Survey (EMDHS 2019) per 1000 live births respectively [9, 10].Globally,tremendous strides were done to reduce neonatal mortality over the past few decades by formulating favorable health policies, and appropriate resource allocation to hasten the achievements of the neonatal survival goals [11–13].By those trials, many countries had reduced neonatal mortality including Ethiopia [14].

Even though, those countries had reduced neonatal mortality, the progress of decline was not impressive in Ethiopia and the contribution of neonatal mortality to under-five death has also increased from 43.3 % to 2016 to 54.5 % in 2019 [9, 10, 15]. As a result, currently, Ethiopiais far from international standards for the prevention of neonatal mortality and it was expected to reduce neonatal mortality as low as 12 per 1000 live in SDG3.2 [16, 17].

However, there were limited institution-basedstudies in southern Ethiopia [18, 19], despite the severity of the problem. Even if, those studies identified some determinants there was inconsistencies in findings. Therefore, this study aimed to provide the representative information on determinants of neonatal mortality among neonates admitted in the neonatal intensive care unit at Dilla University Referral Hospital in Gedeo Zone, Southern Ethiopia.

## Methods

### Study area and period

The study was conducted at Dilla University Referral Hospital (DURH) in Southern Ethiopia, from February, 24 to March 6, 2020. The DURH found in Dilla, the town of Gedeo Zone. It is located due south 359 and 90 km far away from Addis Ababa, and Hawassa, respectively [20]. The Hospital was established in 1977 E.C as a District Hospital. The hospital belongs to the government and its administration was taken by Dilla University since 2005 E.C, hence got the name DURH [21]. Nowadays, the hospital is providing preventive, curative, and rehabilitative services around for 2 million catchment population in different wards [22]. Among those, the neonatal intensive care unit is one, and it has two admission wards for preterm and term neonates’ with a total of 38 beds and 1500 annual neonatal admission with 128 deaths in last year. Concerning the health professionals, three pediatricians, 13 general practitioners and 12 nurses having different profession are providing the health services.

### Study design and population

An institution-based unmatched case-control study was conducted among neonates admitted in the neonatal intensive care unit. All neonates who were admitted in the neonatal intensive care unit and registered as died and alive within the first 28 days in the last one year (2019) period included. Inclusion Criteria for cases, those neonates who admitted and registered as died within the first 28 days of life and have card numbers stated in the logbook and not lost cards with complete chart information were included. Inclusion Criteria for controls, those neonates who admitted and registered as improved within the first 28 days of life and have card numbers stated in the logbook and not lost cards with complete chart information were included. Sample size determination, the sample size was calculated by the StatCalc in Epi-Info version7 statistical software used for sample size determination designed for unmatched case-control study. The following assumptions were considered during sample size calculation; a 95 % level of confidence, power of 80 % and a case to control ratio of one to three (1:3). Considering no single antenatal care service visit (ANC) as an independent exposure variable since, it gave the largest sample size was taken from the study done in Kenya [23]. This gave a total of sample 304 (76 cases and 228 controls. Among public hospitals found in Gedeo Zone, Dilla University Referral Hospital was illegible and selected for this study. Therefore by using the last one-year period medical records, starting from January 31st, 2020 backward to February 1st, 2019 neonatal logbook was reviewed based on their outcome and inclusion criteria.

### Data collection procedures and study variables

The data extraction checklist was adapted after reviewing related journal articles [19, 23–25]by principal investigator. As a data source, a file of admission history, neonatal logbook, history from referral sheet, and delivery summary was used.Based on the medical record numbers, the required numbers of medical record containing cards were drawn out from the card room. Finally, one Bachelor degree of Science (BSc) in midwife and neonatal nurse was collected the data and one health officer supervised data collectors and the collection process was guided by the principal investigator. Dependent variable, neonatal mortality (yes/no).Independent variables Socio-demographic factors: (maternal age, neonatal age, marital status, residence).

Maternal and medical Factors: (parity, ANC follow up, referral status, history of pregnancy-induced hypertension, and Human immune deficiency virus (HIV/AIDS) positive status).

Delivery-related factors: (place of delivery, delivery assistance, mode of delivery.

Newborn factors: (birth weight, preterm, sex of newborn, gestational age, congenital anomalies, sepsis, Activity, Pulse, Grimace, Appearance, and Respiration (APGAR) score, breastfeeding within the first hour, asphyxia, and immediate cry at birth). In an unmatched case-control study of a binary exposure, in which the expected odds ratio deviates from the null (i.e., no effect) and the sum of the number of cases and controls is fixed, 1:1 sampling of cases and controls may yield suboptimal statistical efficiency.

### Data quality assurance techniques, data processing and analysis

The data extraction checklists were prepared in the English language. One-day training was given by the principal investigator on the data extraction checklistand procedure. Before actual data collection, the 5 % of checklists were pre-tested in Sidama Regional State, at Yirgalem General Hospital to check the appropriateness of the checklist. After data collection, a 10 % of collected data was cross checked with medical records and assured its consistency by principal investigator. Finally, the coded data was entered into pass word protected computer statistical software by principal investigator. The data was entered and cleaned for missed values and missed variables using EpiData3.1, then exported and analyzed by Statistical Package for Social Science software (SPSS) version 22. The proportion, frequencies, mean, and standard deviations were computed to compare the exposure status between cases and controls. The degree of association between each independent and dependent variable was assessed by a binary logistic regression analysis model. The variables with p-value < 0.25 during bivariate analysis were selected as candidate variables for multivariate logistic regression analysis model. The Hosmer-Lemeshow goodness of fit test was used to determine model adequacy and the model was adequately fitted for the final analysis with p-value > 0. 581, which was insignificant and that indicates the selected variables were important determinants. The Collinearity between independent variables was checked using variance inflation factor (VIF) and confirmed that, there was no existence of multicollinearity effect. Adjusted odds ratios (AORs) at 95 % CI was used to show the strength of association, and statistical significance was declared at a p-value of < 0.05 as determinant for neonatal mortality.

## Results

### Socio-demographic characteristics

 A total of 304 cases were reviewed (76 cases and 228 controls) from the neonatal intensive care unit in the last one year period (February 1st 2019 to January 31st, 2020), with the overall review rate of 100 %. The mean ages of cases were 5.13 (S.D ± 4.71) days and 7.6 (S.D. ±6.38) days for controls. Also, the mean age of mothers in cases and controls was 24.83(S.D ± 5.75) and 25.09(S.D ± 5.93) years respectively. Around three fourth, 55 (72.4 %) of cases and 126 (55.3 %) of controls were admitted within the first seven days of their age. Besides, the average length of stay in the neonatal intensive care unit for cases and controls was 4.49 and 8.18 days respectively (Table 1).

**Table 1:** Socio-demographic characteristics of mothers and neonates admitted in the neonatal intensive care unit at Dilla University Referral Hospital in Gedeo Zone, Southern, Ethiopia, 2020

Variables	The outcome of the neonates	
	**Case = 76(%)**	**Control = 228(%)**
**Maternal age**		
< 20 years	22 (28.9)	60 (26.3)
20–34 years	46 (60.5)	148 (64.9)
≥ 35 years	8 (10.6)	20 (8.8)
**Age of neonates**		
< 7 days	55 (72.4)	126(55.3)
≥ 7 days	21 (27.6)	102 (44.7)
**Marital status**		
Married	61 (80.2)	197 (86.4)
Single	7 (9.2)	18 (8.0)
Widowed	3 (3.9)	4 (1.7)
Divorced	5 (6.7)	9 (3.9)

### Maternal and medical-related characteristics

In this study, around one third, 23 (30.3 %) of mothers in cases and 34 (14.9 %) of mothers in controls had no single ANC visit during their pregnancy of current neonate. From the mothers having ANC service, thirty seven (69.8 %) of mothers in cases, and 120 (61.8 %) of mothers in controls had ANC visits ranging one up to three. The proportion of mothers who had ANC visit four and above in controls were 74 (38.1 %) which was higher than the proportion of mothers in cases 16 (30.1 %) during the conception of current neonate. The proportions of neonates referred from other health facilities in cases were 53 (69.7 %) which was higher than the proportion of referrals in controls 126 (55.3 %) as illustrated in (Table 2).

**Table 2:** Maternal and medical-related characteristics of mothers of the newborns admitted in the neonatal intensive care unit at Dilla University Referral Hospital in Gedeo Zone, Southern, Ethiopia, 2020

Variables	The outcome of the neonates	
	**Case = 76 (%)**	**Control = 228 (%)**
**Parity**		
1	22 (28.9)	82 (35.9)
2–4	29 (38.1)	97 (42.5)
≥ 5	25 (33.0)	49 (21.6)
**Birth interval**		
< 24 months	43 (56.6)	146 (64.0)
≥ 24 months	33 (43.4)	82 (36.0)
**Mothers Tetanus toxoid (TT) vaccination status**		
Vaccinated	53 (69.7)	194 (85.1)
Not vaccinated	23 (30.3)	34 (14.9)
**Number of TT vaccinated**		
TT1	9 (16.9)	27 (13.9)
TT2+	44 (83.1)	167 (86.1)
**Did mother used iron tablet**		
Yes	53 (69.7)	194 (85.1)
No	23 (30.3)	34 (14.9)
**Number of Fe tablets used**		
Complete recommended	11 (20.7)	41 (21.1)
Not complete recommended	42 (79.3)	153 (78.9)
**Referral status**		
Not referred	23 (30.3)	102 (44.7)
Referred	53 (69.7)	126 (55.3)
**Pregnancy induced hypertension**		
Yes	7(9.3)	17 (7.5)
No	69 (90.7)	211 (92.5)
**Postnatal care receiving status**		
Received	37(48.7)	124(54.4)
No received	39(51.3)	104(45.6)

### The description of delivery-related characteristics

This study found 27 (35.5 %) of mothers in cases and 99 (43.4 %) of mothers in controls gave birth at the hospital. The remaining, 19 (25 %) and 30 (39.5 %) of mothers in cases and 35 (15.4 %) and 94 (41.2 %) of mothers in controls, were from home and health center deliveries respectively. The proportion of assisted vaginal delivery was, 17 (22.4 %) in cases which was higher than the proportion in controls 26 (11.4 %). The proportion of cesarean section was, 7 (9.2 %) in cases and 30 (13.2 %) in controls. Also, the mothers whose labor was not followed by partograph sheet in cases were 34 (59.6 %) which was much higher than the proportion in controls 30 (15.5 %) as indicated in (Table 3).

**Table 3:** The description of delivery-related characteristics of mothers of newborns admitted in the neonatal intensive care unit at Dilla University Referral Hospital in Gedeo, Southern, Ethiopia, 2020

Variables	The outcome of the neonates	
	**Case = 76(%)**	**Control = 228(%)**
**Mode of delivery**		
Spontaneous vaginal delivery	52(68.4)	172(75.4)
**Partograph**		
Yes	23 (40.4)	163 (84.5)
**Duration of labor**		
≤ 11 h	8 (34.7)	60 (36.8)
12–18 h	6 (26.2)	58 (35.8)
≥ 19 h	9 (39.1)	45 (27.4)

### The description of neonatal related characteristics

More than half of cases, 48 (63.2 %) and 56 (24.6 %) of controls, were below 37 week of gestational age respectively. The proportion of asphyxia in cases were 35 (61.4 %) which was higher than the proportion of asphyxia, 67 (34.7 %) in controls. Regarding sepsis, more than half, 42 (55.3 %) of cases and 57 (25 %) of controls were positive for neonatal sepsis. Also, the proportion of early initiated breastfed within the first hour after delivery in controls was 156 (68.4 %) which was much higher than the proportion in cases 32 (42.1 %). Similarly, the proportion of neonates who cried immediately at birth in controls was 118 (61.1 %) which was higher than the event in cases, 28 (49.1 %) and the remaining variables were indicated (Table 4).

**Table 4:** The description of neonate related characteristics who admitted in the neonatal intensive care unit at Dilla University Referral Hospital in Gedeo Zone, Southern, Ethiopia, 2020

Variables	The outcome of the neonates	
	**Case = 76(%)**	**Control = 228(%)**
**Weight of the neonates**		
< 2500 g	54(71.1)	70 (30.7)
≥ 2500 g	22 (28.9)	158 (69.3)
**Gestational weeks**		
< 37 Wks	48 (63.2)	56 (24.6)
≥ 37 Wks	28 (36.8)	172(75.4)
**Respiration rate of the neonates**		
31–60 Breath/min	37 (48.7)	136 (59.6)
≥ 61 Breath/min	31 (40.8)	77 (33.8)
≤ 30 Breath/min	8 (10.5)	15 (6.6)
**Asphyxia**		
Yes	35 (61.5)	67 (34.7)
No	22 (38.5)	126 (65.3)
**Temperature**		
< 35.5 °C	17 (22.4)	32 (14.1)
35.5–37.5 °C	48 (63.1)	156 (68.4)
≥ 37.6 °C	11 (14.5)	40 (17.5)
**Sepsis**		
Yes	42 (55.3)	57 (25)
No	34(44.7)	171(75)
**Breastfed within 1**^**St**^**hr after birth**		
Yes	32 (42.1)	156 (68.4)
No	44 (57.9)	72 (31.6)
**Neonate fed colostrums**		
Yes	46 (60.5)	164 (71.9)
No	30 (39.5)	64 (28.1)
**Sex of neonate**		
Male	41(53.9)	118 (51.8)
Female	35 (46.1)	110 (48.2)
**APGAR at 1st min**		
< 7	46 (80.7)	153 (79.3)
≥ 7	11 (19.3)	40 (20.7)
**APGAR at 5th min**		
< 7	18 (31.6)	46 (23.8)
≥ 7	39 (68.4)	147 (76.2)
**Cry immediately at birth**		
Yes	28 (49.1)	118 (61.1)
No	29 (50.9)	75 (38.9)

### The weight of neonates

**Fig. 1:**
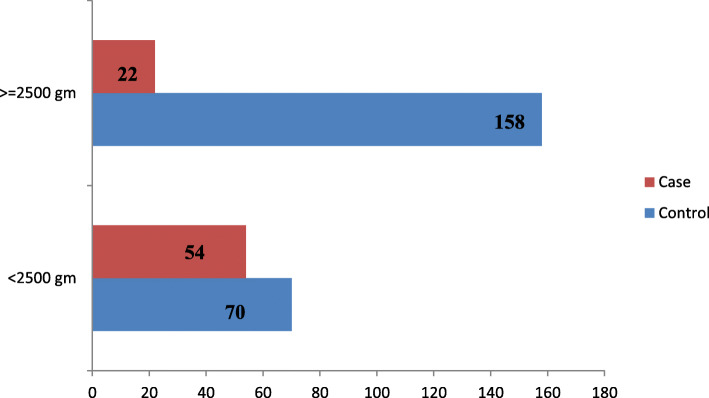
Birth weight of cases and controls as determinants to neonatal mortality among newborns admitted in the neonatal intensive care unit at Dilla University Referral Hospital in Gedeo Zone, Southern, Ethiopia, 2020

The current study found, the proportion of low birth weight (< 2500 g) in cases was 54 (71.1 %), which was much higher than that of proportion in controls 70 (30.7 %) (Fig. 1).

### Determinants associated with neonatal mortality

In bivariate analysis, ANC visit, referral status, mode of delivery, gestational age, the weight of newborn, asphyxia, sepsis, statusof breastfed within the first hour, fed colostrum and the ability of neonate to cry at birth were determinants associated with neonatal mortality. In multivariate analysis, referral status, gestational age, the weight of newborns, breastfeedingand sepsis had remained to have an association with neonatal mortality. Those neonates referred from other health institution were 2.43 timesmore likely to die during neonatal period compared to those neonates who were delivered and had follow up in the study hospital[AORs = 2.43, 95 % CI (1.14, 5.22)].Concerninggestational age, those neonates delivered before 37 weeks of gestational agewere 2.50 times more likely to die within the first 28 days of life compared to those neonates delivered at ≥ 37 weeks of gestational age[AORs = 2.50, 95 % CI (1.12, 5.58)].Similarly, those neonates weighing less than 2500 g at birth were 2.44times more likely to die compared with the neonatesweighing greater than or equal to 2500 g at birth [AORs = 2.44, 95 %CI (1.13, 5.28)]. Besides, those neonates who did not breastfeed immediately within the first hour after delivery were5.24 times more likely to die compared to those neonates who fed breast immediately at birth [AORs = 5.24, 95 %CI (2.42, 11.37)].Moreover, those neonates who were positive for sepsis diagnosis were 2.45 times more likely to experience neonatal mortality compared to the neonates who were not positive for sepsis [AORs = 2.45, 95 %CI (1.11, 5.41)] in (Table 5).

**Table 5:** Bivariate and multivariate logistic regression analysis on determinants associated with neonatal mortality among newborns admitted in the neonatal intensive care unit at Dilla University Referral Hospital in Gedeo Zone, Southern, Ethiopia, 2020

Exposure variables	Cases	Controls	CORs with 95 % CI	AORs with 95 % CI
**Number of ANC**				
≥ 4 visit	16 (21.10)	74 (32.5)	1	1
2–3 visit	37 (48.7)	120 (52.6)	1.43 (0.74, 2.74)	1.03 (0.43, 2.47)
No single visit	23 (30.3)	34 (14.9)	3.13** (1.47, 6.67)	2.4 (0.90, 6.39)
**Referral status**				
Not referred	23 (30.3)	102 (44.7)	1	1
Referred	53 (69.7)	126 (55.3)	0.53*(1.07, 3.25)	2.43 (1.14, 5.22)*
**Mode of delivery**				
SVD	52 (68.4)	172 (75.4)	1	1
AVD	17 (22.4)	26 (11.4)	2.16*(1.09, 4.29)	1.99 (0.83, 4.75)
CS	7 (9.2)	30 (13.2)	0.77 (0.32, 1.86)	0.87 (0.28, 2.67)
**Gestational Age**				
< 37 Wks	48 (63.2)	56 (24.6)	5.26*** (2.87, 8.65)	2.50 (1.12, 5.58)*
≥ 37 Wks	28(36.8)	172 (75.4)	1	1
**Weight of neonate**				
< 2500 g	54(71.1)	70 (30.7)	5.54*** (3.13, 9.79)	2.44(1.13, 5.28)*
≥ 2500 g	22(28.9)	158 (69.3)	1	1
**Asphyxia**				
Yes	35 (61.4)	67 (34.7)	2.99** (1.20, 3.49)	1.97(0.99, 3.88)
No	22 (38.5)	126 (65.2)	1	1
**Sepsis**				
Yes	42 (55.3)	57 (25)	3.70*** (2.11, 6.22)	2.45 (1.11, 5.41)*
No	34 (44.7)	171 (75)	1	1
**Breast Feed within 1**^**St**^**hour**				
Yes	32 (42.1)	156 (68.4)	1	1
No	44 (57.9)	72 (31.6)	0.33***(1.66, 4.81)	5.24(2.42, 11.37)***

*p < 0.05,**p < 0.01, ***p < 0.001, *SVD* Spontaneous vaginal delivery, *AVD *assisted vaginal delivery, *CS* caesarean section, *g* gram

## Discussion

This study assessed the determinants of neonatal mortality and identified the determinants like; referrals from other health institution, neonates delivered before 37 weeks of gestational age, newborns with birth weight less than 2500 g, neonates who did not feed breast within the first hour after delivery and neonates who were positive for sepsis, as the statistical significant determinants associated with neonatal mortality. Hence, the study findings have implications for the health services targeted to reduce neonatal mortality in the study hospital.

The referrals from other health institution were found to be significantly associated with the occurrence of neonatal mortality. Particularly, the neonates referred from other health institution were 2.43 times more likely to die when compared to the neonates who had no referral. This finding is consistent with the study conducted in Woliata, Ethiopia reported that cases referred from other health institution was 7.32 times more likely to end up in neonatal mortality when compared to the neonates from the study hospital [18]. This finding may be due to birth trauma, infection, delayed action in the health institution and lack of immediate newborn care [12].

The neonates delivered before 37 weeks of gestational age were measured as having a significant association with the event of neonatal mortality. Specifically, those newborns with a gestational age less than 37 weeks were 2.50 times more likely to pass away when compared to neonates who delivered at gestational age of 37 weeks and above.This finding is in line with the result from the study in Rwanda showed newborns with a gestational age less than 37 weeks were 3.1 times more likely to die than their counterparts [26]. Similarly, this result is in agreement with the study in Kenya revealed that cases with gestational age < 37 weeks were 7.0 times more likely to die when compared to controls with gestational age of 37 weeks and above[23]. Likewise the study in Adama, Ethiopia also showed consistent finding with this study in which preterm neonates were 3.3 times more likely to die compared to term neonates [27]. This result might be due to immaturity of respiratory and cardiovascular organs, vulnerability to infection, hypothermia, and lack of skilled medical care during intrapartum, and postpartum period [3, 4].

In this study, the birth weight of newborn less than 2500 g was significantly associated with the event of neonatal mortality. Especially, those neonates weighing less than 2500 g were 2.44 times more likely to die when compared to the neonates weighing 2500 g and above at birth. The current finding is consistent with the report of study from Jimma, Ethiopia which showed cases with low birth weight was 1.54 times more likely to die when compared to their counterparts [25]. Similarly, the study conducted in Adama, Ethiopia illustrated comparable finding with this study in which neonates with birth weight < 2500 g were 1.6 times more likely to die than the neonates with birth weight ≥ 2500 g [27]. Likewise, this result also concur with the study in Wolaita, Ethiopia found low birth weight were 9.0 times more likely to die when compared to newborns with normal birth weight [18]. This finding might be due to birth defect, infections, hypothermia, lack of medical care during intrapartum and postnatal period. Also it might be due to inability to adapt the external environment [3, 7].

This study also found that neonates who didn’t feed breastwithin the first hour after delivery was positively associated with the experience of neonatal mortality. Specifically, neonates who didn’t feed breast milk immediately were 5.24 times more likely to die when compared to those neonates who were breastfed immediately at birth. This finding is in line with the study conducted in Indonesia showed, cases who didn’t feed breast early were 10.46 times more likely to die when compared to their counterparts [28]. Similarly, this finding is in consistent with the study in Amhara, Ethiopia reported neonates who didn’t feed breast within the firsthour of delivery were 23.48 times more likely to die than their counterparts [29]. Likewise, a study in Woliata Ethiopia found comparable finding in which neonates who were not breastfed within first hour were 2.6 times more likely to die than those who breastfed immediately after birth [19]. This finding might be due to lack of protective effect of early initiated breast milk, and increased susceptibility to gastroenteritis, common newborn infections and diseases, as indicated in different reports [7, 12, 13].

Besides, in current study those neonates who were positive for sepsis diagnosis was significantly associated with the event of neonatal mortality. Uniquely, those neonates who were positive for sepsis were 2.45 times more likely to diewhencompared to the neonates who were not positive for sepsis. This finding is concurring with the study report from Adama Ethiopia revealed cases with positive for sepsis were 2.4 times more likely to die when compared to their counterparts [27]. Similarly, this finding in line with the study in MizanTepi, Ethiopia showed cases positive for sepsis were higher risk of death when compared to the neonates not positive for sepsis [24]. This result might be due to the extreme response to an infection, tissue damage, organ failure and lack of immediate medical care [7].

In current study ANC receiving status was not significantly associated with the occurrence of neonatal mortality in multivariate analysis. This finding is contradicting with the studies conducted in Rwanda, Democratic Republic of Congo (DRC), Kenya, Ethiopia (Amhara, and Wolaita) [19, 23, 26, 29, 30], showed those neonates delivered from mothers who had no or incomplete ANC visit were more likely to die than neonates from mothers who have followed ANC visit four and above. This discrepancy might be due to methodological difference and inconsistency of records.

The strength of current study compared one case with three controls to increase the representativeness of the sample, and crosschecked a 10 % of collected data with medical records and assured consistency by principal investigator. The major limitations of this study are its design and selection bias. Only neonates assessed who used healthcare services in the DURH, were included in this study. Another limitation is related to estimation methods. They generally do not allow calculation of incidence (absolute risk). This study used secondary data as a source of information which was not specific and there were difficulties to measure some variables. Further, the collected data was from a single institution, so it may have some drawbacks, regarding population under the study. Also, this study failed to measure the status of neonates who discharged alive before 28 days after admission. Hence, this may decrease the external validity of the study.

## Conclusions

Results from this study suggest that the referral status, gestational age below 37 weeks, birth weight less than 2500 g, neonates who did not feed breastwithin the first hour after delivery, and neonates positive for sepsis diagnosis were measured as the significant independent determinants to neonatal. This study shows that neonatal intensive care unit service should be strengthened in Dilla University Referral Hospital; targeting neonate aged below 28 days. Most of the determinants may be prevented and minimized by strengthening referral linkage, improving intrapartum and postpartum care. Further research is needed in order to better understand reasons for neonatal mortality. Further, those who are interested should conduct an institution based incidence study to see the sequence of event.

## Data Availability

All data are available within manuscript and it will be upon reasonable request from the corresponding author.

## Abbreviations

AOR: Adjusted Odds Ratio
APGAR: Activity, Pulse, Grimace, Appearance, and Respiration
COR: Crude Odds Ratio
DHS: Demographic Health Survey
DRC: Democratic Republic of Congo
DURH: Dilla University Referral Hospital
EDHS: Ethiopian Demographic Health Survey
EMDHS: Ethiopian Min Demographic Health Survey
MOH: Ministry of Health
LBW: Low Birth Weight
NICU: Neonatal Intensive Care Unit
NMR: Neonatal Mortality Rate
SDG: Sustainable Development Goal
